# Research on Electro-Hydraulic Servo Resonance Technology

**DOI:** 10.3390/s24123992

**Published:** 2024-06-20

**Authors:** Xiuguang Yang, Peng Liu, Hongwei Zhao

**Affiliations:** 1 School of Mechanical and Aerospace Engineering, Jilin University, Changchun 130025, China; yangxg0312@163.com (X.Y.);; 2 SinoTest Equipment Co., Ltd., Changchun 130103, China

**Keywords:** electro-hydraulic servo, resonance, simulation analysis, mode

## Abstract

In this paper, an electro-hydraulic servo resonance technology is proposed to meet the loading requirements of a high-frequency sound fatigue test for large tonnage. First of all, we analyze the static and dynamic loading structure of electro-hydraulic servo vibration and establish the vibration equation of the system. Additionally, the modal and vibration characteristics of the system are analyzed by simulation, which verified the feasibility of the proposed electro-hydraulic servo resonant loading technology. Finally, the influence of various factors such as sample stiffness, lead screw stiffness, class II spring stiffness, class II weight mass, lower beam mass, and upper beam mass on the natural frequency and amplification coefficient of the system is analyzed. In this paper, a new technology is proposed to provide theoretical support for the research and development of large-tonnage high-frequency noise fatigue testing equipment.

## 1. Introduction

With the advancement of society and the progress in science and technology, the structural size of major equipment has become increasingly large-scale [1,2,3]. However, due to the size effect, small-scale mechanical tests on smooth specimens are no longer sufficient to comprehensively evaluate the mechanical properties of full-size equipment. Therefore, it is necessary to conduct fatigue tests on large-tonnage loads of full-size materials and components [4,5]. This is particularly important for high-end equipment such as high-speed trains and engines, which have complex structures and require engineering components to have a fatigue life of 10^9^~10^12^ cycles [6,7]. In order to meet these requirements, there is a need to improve the working frequency and output load of fatigue tests. This will help to shorten the fatigue test cycle and save significant human, material, and financial resources. The full-size large-tonnage and higher frequency test requirements have forced the industry to continue to put forward new test technology requirements.

The fatigue test is crucial in aerospace engineering, civil engineering, and mechanical engineering. In the deep-sea field, the fatigue test of large-tonnage structures is also suitable for studying the fatigue crack caused by the fluctuation of the fluid pressure in the pipeline under high pressure in marine engineering [8,9,10,11]. The performance of the fatigue testing machine is primarily measured by its working frequency, loading capacity, and energy consumption [12]. An ideal fatigue testing machine should be capable of loading across a wide frequency and load range while consuming less energy [13]. As the demand for test technology applications grows, enhancing the working frequency and loading capacity of testing machines and reducing energy consumption has become the primary focus of future studies.

At present, there are two main types of fatigue testing machines: the electro-hydraulic servo fatigue testing machine [14,15,16] and the electromagnetic resonance fatigue testing machine [17]. The electro-hydraulic servo fatigue testing machine is capable of handling large tonnage and various stress types, but it has a low loading frequency and consumes a large amount of energy. On the other hand, the electromagnetic resonance fatigue testing machine can achieve high-frequency loading, energy efficiency, and environmental friendliness, but it has limitations in terms of stress load and loading type. It is evident that neither of these fatigue test machines can fully meet the current testing research requirements.

Therefore, this paper proposes an electro-hydraulic servo resonant loading technology. It designs an electro-hydraulic servo harmonic loading structure, analyzes the vibration characteristics of the system, verifies the functional principle of the electro-hydraulic servo resonant system through simulation analysis, and analyzes the two key design parameters of the system: natural frequency and magnification.

## 2. Structural Principle and Vibration Analysis

### 2.1. Structural Principle

**Figure 1:**
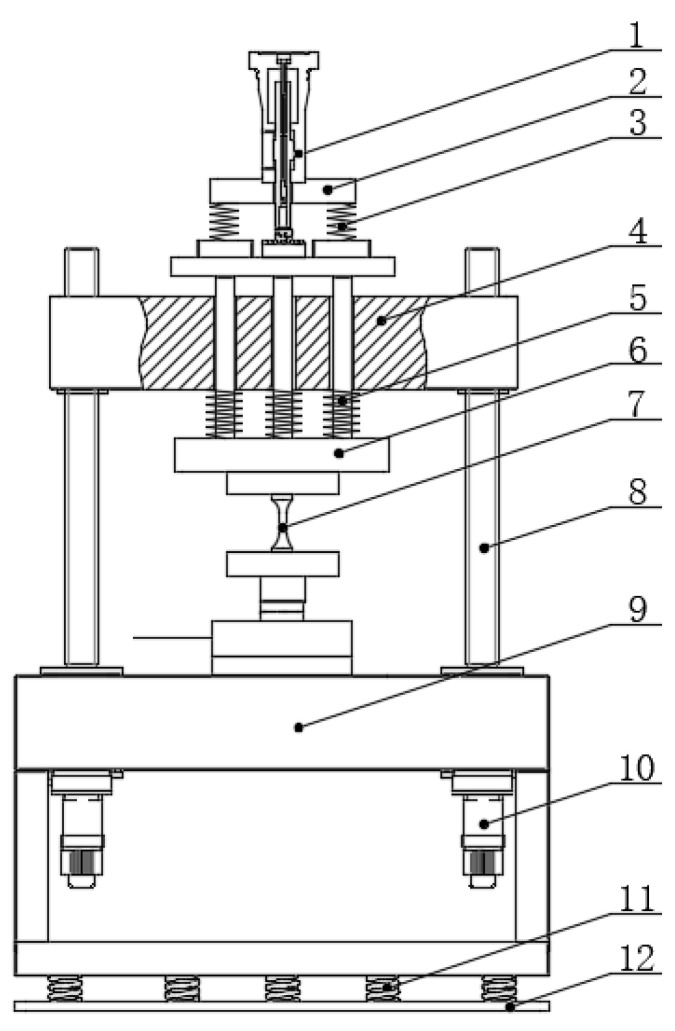
Structure diagram of the electro-hydraulic servo resonant system (1—hydrostatic servo actuator, 2—class I weight, 3—class I spring, 4—upper beam, 5—class II spring, 6—class II weight, 7—sample, 8—lead screw drive unit, 9—lower beam, 10—servo motor unit, 11—shock absorber spring, and 12—bottom plate).

The structure principle of the electro-hydraulic servo resonance loading technology is shown in Figure 1. The components of the system include the following: hydrostatic servo actuator, class I weight, class I spring, upper beam, class II spring, class II weight, sample, lead screw drive unit, lower beam, servo motor unit, shock absorber spring, and base plate. These components work together to apply both alternating load and average load on the sample. The excitation loading is achieved through the hydrostatic servo actuator unit, which generates high-frequency excitation and outputs a small alternating load. The resonance loading is accomplished by the resonance system composed of a class I spring, class II spring, class I weight, and class II weight. This system generates high frequency by amplifying the high frequency, small load from the hydrostatic servo actuator, and large load for the sample. The class I spring and class II spring also serve the purpose of energy storage and release, reducing the energy consumption of the test load. The mean loading is achieved through the servo motor unit, lead screw drive unit, and upper beam, enabling the application of average load on the sample. And a dual servo motor synchronous closed-loop control is employed. Additionally, damping springs are incorporated to minimize the impact of system vibration on the surrounding environment.

### 2.2. Electro-Hydraulic Servo Excitation Equivalent Stiffness

**Figure 2:**
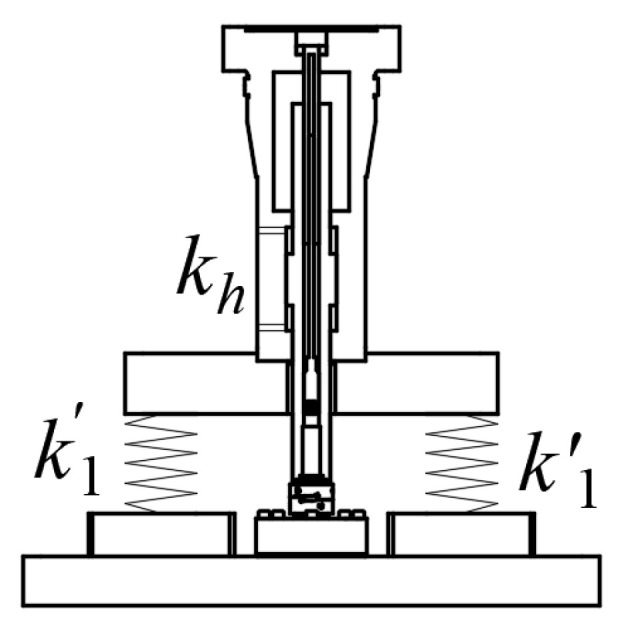
Electrohydraulic servo excitation structure.

The primary function of electro-hydraulic servo excitation is to provide high-frequency sound and small load excitation force. The structure of electro-hydraulic servo excitation is shown in Figure 2, consisting of a hydrostatic servo actuator and a class I spring. The hydrostatic servo actuator is equipped with a displacement sensor and a force sensor for closed-loop control.

The sealing and guiding of the hydrostatic support servo actuator are all realized by the hydrostatic bearing, and the journal lining of each hydrostatic bearing has an oil chamber that communicates with each other; when the piston rod is subjected to a transverse load, the hydraulic pressure in the two opposing bearing oil chambers will be redistributed, so that the piston rod returns to the position of the center of the cylinder block, and this working mode dynamically keeps the position that the piston rod is in the center of the cylinder block, and the coulomb friction force does not occur all the time. Due to the low friction of the static pressure servo actuator, it can be assumed to be an ideal friction-free and leak-free servo actuator. When working, the two working chambers are filled with hydraulic oil and the compressibility of hydraulic oil. Then, the hydraulic spring stiffness $k_{h}$ is shown in Equation (1): $\beta_{e}$ indicates bulk elastic modulus of liquid, Pa; $V_{t}$ indicates initial volume of oil return chamber, m^3^; A indicates effective piston area, m^2^; and $2k_{1}$ indicates electro-hydraulic servo excitation equivalent stiffness.

(1) $$k_{h}=\frac{4\beta_{e}A^{2}}{V_{t}}$$

(2) $$2k_{1}=2k_{1}^{\prime }+k_{h}$$

The equivalent elastic stiffness of the electro-hydraulic servo excitation module is composed of the hydraulic spring stiffness and the spring stiffness of the class I spring in parallel, that is, according to the hydraulic spring stiffness calculation formula shown in Equation (2).

### 2.3. Resonant Vibration

**Figure 3:**
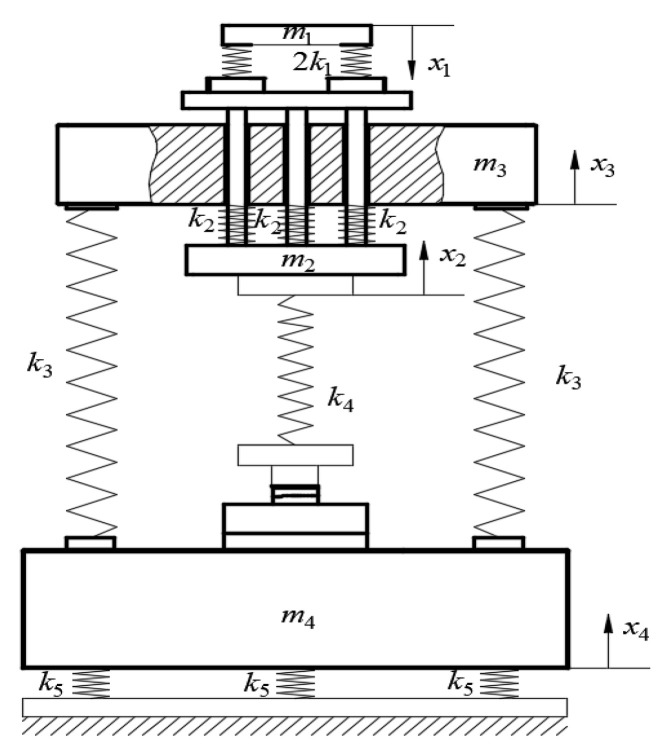
Resonant vibration model.

The function of resonant vibration is to convert the high-frequency sound and small load excitation force generated by the electro-hydraulic servo excitation module into high-frequency sound and large load test force in order to apply the load to the sample [18]. The resonant vibration system can be simplified as a mass-spring system, as shown in Figure 3. It consists of the following components: $m_{1}$—hydrostatic servo actuator cylinder and class I weights; class II spring, which is composed of three springs with a spring stiffness of $k_{2}$; lead screw and sample, considered as elastomers with spring stiffness of $k_{3}$ and $k_{4}$; damping spring, which consists of six springs with a spring stiffness of $k_{5}$; $m_{2}$—class II weight; $m_{3}$—upper beam mass; and $m_{4}$—lower beam mass. The resulting vibration displacements of $m_{1}$, $m_{2}$, $m_{3}$, and $m_{4}$ are denoted as $x_{1}$, $x_{2}$, $x_{3}$, and $x_{4}$, respectively.

**Figure 4:**
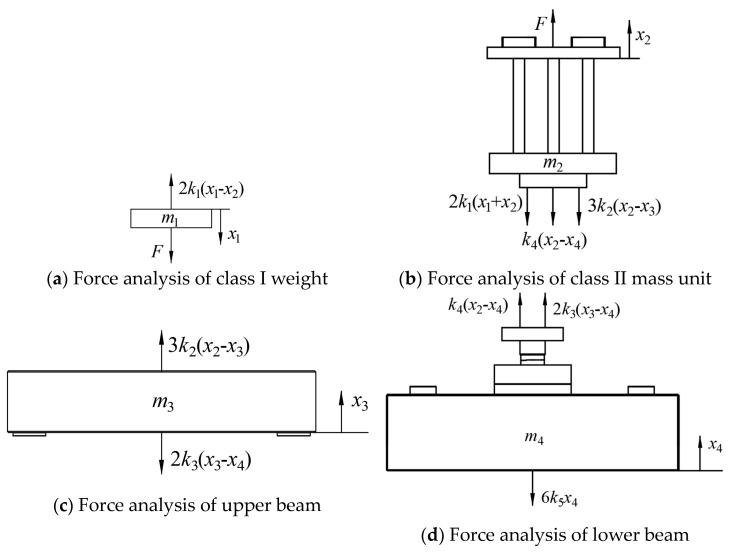
Force analysis of vibration unit.

Force analysis was carried out on each part, as shown in Figure 4.

The vibration is shown in Equation (3).

(3) $$\left\{ \begin{array}{l} F-2k_{1}(x_{1}+x_{2})=m_{1}{\ddot{x}}_{1}\\ F-2k_{1}(x_{1}+x_{2})-k_{4}x_{2}-3k_{2}(x_{2}-x_{3})=m_{2}{\ddot{x}}_{2}\\ 3k_{2}(x_{2}-x_{3})-2k_{3}(x_{3}-x_{4})=m_{3}{\ddot{x}}_{3}\\ k_{4}(x_{2}-x_{4})+2k_{3}(x_{3}-x_{4})-6k_{5}x_{4}=m_{4}{\ddot{x}}_{4} \end{array}\right.$$

Thus, Equation (4) is obtained. [*m*] and [*k*] are, respectively, the mass matrix and the stiffness matrix, as shown in Equations (5) and (6).

(4) $$\left[m\right]\left\{\begin{array}{c} {\ddot{x}}_{1}\\ {\ddot{x}}_{2}\\ {\ddot{x}}_{3}\\ {\ddot{x}}_{4} \end{array}\right\}+\left[k\right]\left\{\begin{array}{c} x_{1}\\ x_{2}\\ x_{3}\\ x_{4} \end{array}\right\}=\left\{\begin{array}{c} F\\ F\\ 0\\ 0 \end{array}\right\}$$

(5) $$\left[m\right]=\left[\begin{array}{cccc} m_{1} & 0 & 0 & 0\\ 0 & m_{2} & 0 & 0\\ 0 & 0 & m_{3} & 0\\ 0 & 0 & 0 & m_{4} \end{array}\right]$$

(6) $$\left[k\right]=\left[\begin{array}{cccc} 2k_{1} & 2k_{1} & 0 & 0\\ 2k_{1} & 2k_{1}+3k_{3}+k_{4} & -3k_{2} & -k_{4}\\ 0 & -3k_{2} & 3k_{2}+2k_{3} & -2k_{3}\\ 0 & -k_{4} & -2k_{3} & 2k_{3}+k_{4}+6k_{5} \end{array}\right]$$

According to the mass-spring vibration theory, $m_{1}$, $m_{2}$, $m_{3}$, and $m_{4}$ move in unison, the second-order linear homogeneous differential system with constant coefficients has a synchronous solution. This paper assumes that Equation (7) holds. $u_{j}$ indicates the set of constants.

(7) $$x_{j}(t)=u_{j}f(t)\;\;j=1,\;2,\;3,\;4$$

And f(t) is a real function that depends on time. It is the same for all coordinates, as shown in Equation (8). *const* indicates real constant.

(8) $$\frac{x_{j}(t)}{x_{i}(t)}=\frac{u_{j}}{u_{i}}=const\;\;\;\;\;\;\;\;\;i,j=1,\;2,\;3,\;4$$

That is, the ratio of displacements in any two coordinates is a constant independent of time, which indicates that the coordinates are moving in proportion. Because the damping of the system is ignored, it is also free vibration without external excitation, and it is a conservative system. The equation is the motion equation of the resonant subsystem, and its solution is shown in Equation (9). C indicates constant; ω indicates simple harmonic motion frequency, ωt=λ; and φ indicates initial phase.

(9) f(t)=Ccos(ωt−ϕ)

Bring Equation (7) into Equation (4) to obtain Equation (10).

(10) $$(\left[k\right]-\omega^{2}\left[m\right])\left\{u\right\}=0$$

This is a four-element linear homogeneous system of equations with respect to {u}, and the necessary and sufficient condition for this system to have a nonzero solution is that its coefficient determinant is equal to zero; the solution formulas for ω are shown in Equations (11) and (12).

(11) $$\Delta (\omega^{2})=\left|k_{ij}-\omega^{2}m_{ij}\right|=0$$

(12) $$\Delta (\omega^{2})=\left|\begin{array}{cccc} 2k_{1}-\omega^{2}m_{1} & 2k_{1} & 0 & 0\\ 2k_{1} & 2k_{1}+3k_{3}+k_{4}-\omega^{2}m_{2} & -3k_{3} & -k_{4}\\ 0 & -3k_{2} & 3k_{2}+2k_{3}-\omega^{2}m_{3} & -2k_{3}\\ 0 & -k_{4} & -2k_{3} & 2k_{3}+k_{4}+6k_{5}-\omega^{2}m_{4} \end{array}\right|=0$$

The system frequency equation of ω^2^ can be obtained by expanding it, as shown in Equation (13), where a_1_, a_2_, a_3_, and a_4_ are the functions of k_1_~k_5_ and m_1_~m_4_.

(13) $$\omega^{8}+a_{1}\omega^{6}+a_{2}\omega^{4}+a_{3}\omega^{2}+a_{4}=0$$

Since both the mass matrix and the stiffness matrix are positive definite real symmetric matrices, the four roots of the system frequency equation are characteristic roots of positive real numbers $w_{j}(j=1,2,3,4)$, which correspond to the four natural frequencies of the system. The corresponding modal vector {u(j)} can be obtained by substituting each characteristic root into the equation, and the natural frequency $\omega_{j}(j=1,2,3,4)$ and modal vector {u(j)} constitute the j order natural mode of the system. The mode equation is shown in Equation (14). The system is a 4-degree-of-freedom system with 4 synchronous motions, each of which is simple harmonic motion but with different frequencies.

(14) $$\left\{x{\left(t\right)}^{\left(j\right)}\right\}=\left\{u^{\left(j\right)}\right\}\cos(\omega_{j}t-\phi_{j}),\;\;\;\;\;\;\;\;\;\;\;\;\;\;j=1,2,3,4$$

## 3. System Simulation Analysis

### 3.1. Simulation Model and Parameter Setting

**Figure 5:**
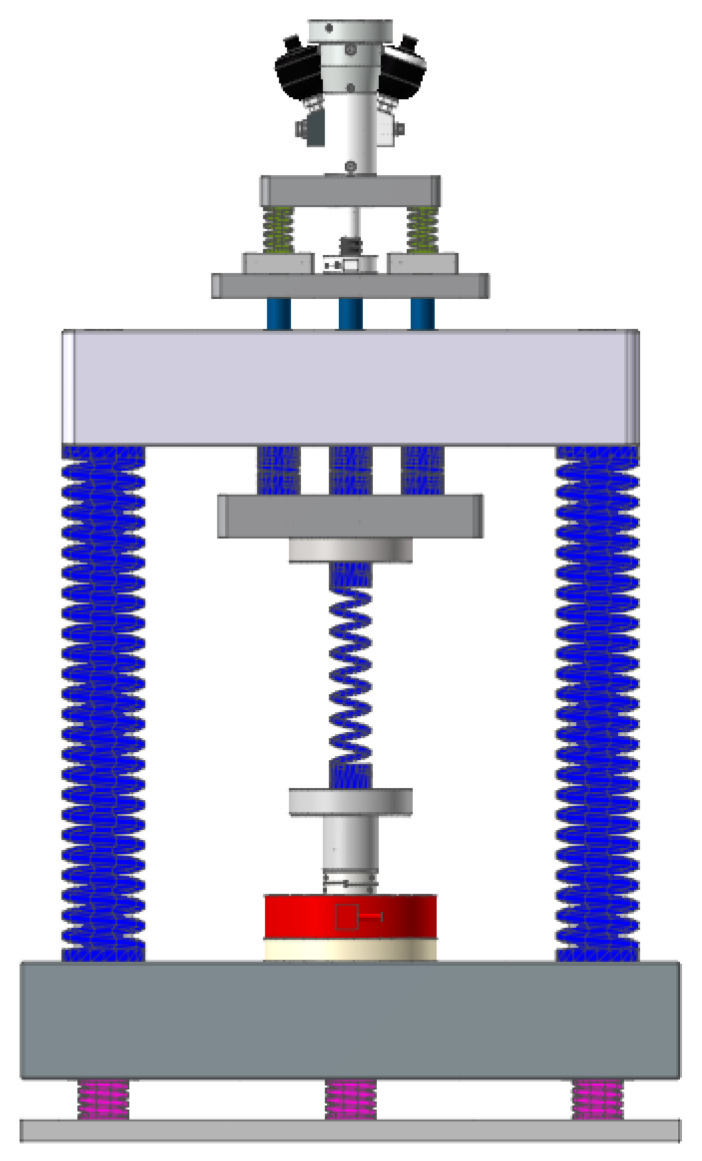
Simulation model.

The simulation model of the electro-hydraulic servo resonant loading system was established by using Recurdyn, V9R5 a multilift dynamic simulation software, as shown in Figure 5, and the parameter settings of each part are shown in Table 1.

### 3.2. Modal Analysis

The eigenvalue analysis tool in Recurdyn simulation software was used to analyze the eigenvalue of the system. The eigenvalue of the system is shown in Table 2.

Below are the modal vectors at different natural frequencies:


$$\left[\{u^{\left(1\right)}\}=\{\begin{array}{c} -1.039\\ -0.999\\ -1.000\\ -0.999 \end{array}\}\right]\;\left\{u^{\left(2\right)}\}=\{\begin{array}{c} 0.868\\ -0.124\\ -0.114\\ -0.123 \end{array}\right\}\;\left\{u^{\left(3\right)}\}=\{\begin{array}{c} 0.012\\ 0.264\\ -0.780\\ 0.184 \end{array}\right\}\;\left\{u^{\left(4\right)}\}=\{\begin{array}{c} 0.0002\\ -0.633\\ -0.052\\ 0.251 \end{array}\right\}$$

Note: The modal vectors from top to bottom are the modes of class I weights, upper beams, class II weights, and lower beams, respectively.

**Figure 6:**
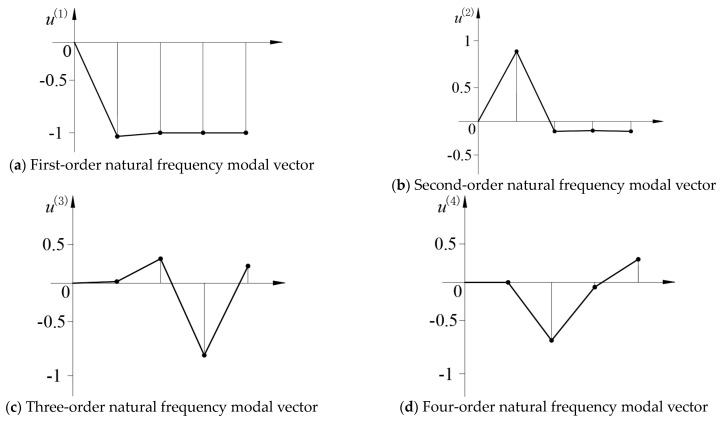
Modal vector.

The modal vector is represented by coordinates, as shown in Figure 6.

The first-order natural frequency is 4.69 Hz, the direction of the modal vector of the I class weight, the upper beam, the II class weight, and the lower beam is the same, and the amplitude is basically equal, mainly the shock absorber spring and the resonance point of the lower beam.

The second-order natural frequency is 25.86 Hz, and the amplitude of the modal vector of the I grade weight is the largest, and the amplitude of the modal vector of the upper beam, the II grade weight, and the lower beam are small and basically the same, mainly the static pressure actuator cylinder, the I grade weight, and the I spring resonance point.

The third-order natural frequency is 198.62 Hz, and the amplitude of the modal vector of the II weight is the largest, and the amplitude of the modal vector of the upper beam, the I weight, and the lower beam is basically the same, mainly the static pressure actuator piston rod, the II weight, and the II spring resonance point.

The fourth-order natural frequency is 386.54 Hz, and the amplitude of the modal vector of the upper beam is the largest, and the amplitude of the modal vector of the II level weight, the I level weight, and the lower beam is basically the same, mainly the lead screw, the upper beam, and the sample resonance point.

To realize the resonant loading of the sample, the excitation frequency should be the third-order natural frequency, and the first-order natural frequency should be controlled below 5 Hz, which is mainly used for shock absorption. The second-order natural frequency should avoid interfering with the test frequency, which should be much lower than the test frequency. The fourth-order natural frequency should avoid interference with the test frequency, which is much higher than the test frequency.

### 3.3. Vibration Analysis

**Figure 7:**
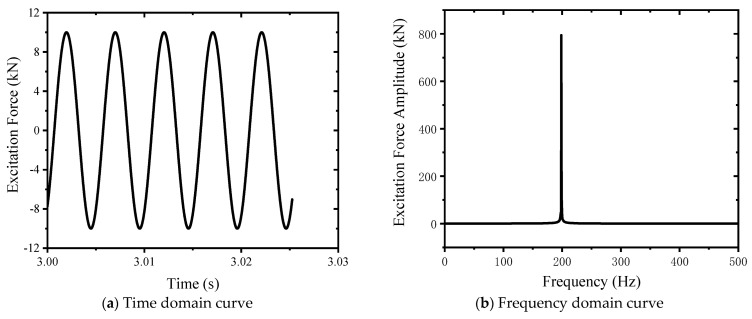
Exciting force curve.

Dynamic/kinematic analysis tool in Recurdyn simulation software was used to carry out vibration analysis of the system. According to the modal analysis results, the excited force is applied to the static pressure support actuator according to the third-order natural frequency. The waveform of the excited force is a sine wave, the half amplitude is 10 kN, the frequency is 198.62 Hz, and the average value is 0 kN. The excited force curve is shown in Figure 7. This meets the design requirements.

#### 3.3.1. Enlargement Factor

**Figure 8:**
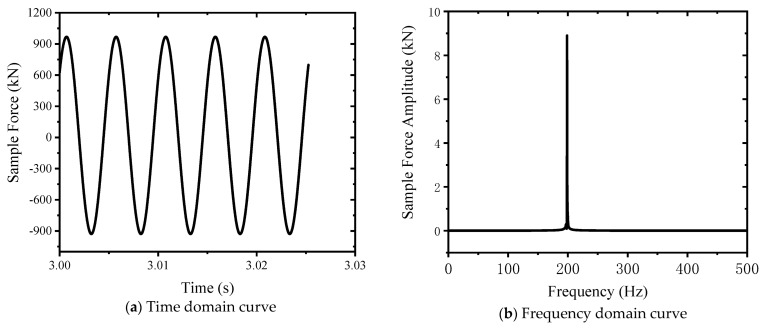
Specimen force curve.

The magnification ratio is one of the key parameters of an electro-hydraulic servo resonant system; it is the ratio of sample force to exciting force, which reflects the amplification ability of exciting force through the resonant system. Through the analysis of the resonant force subjected to the test, the waveform of the sample force is a sine wave, the frequency is 198.62 Hz, the peak value is 967.7 kN, and the valley value is −928.8 kN, and the half amplitude of the sample force is (967.7 + 928.8)/2 = 948.25 kN, as shown in Figure 8.

Note: Although the mean value of the applied exciting force is 0 kN, due to the influence of gravity on the components, such as class I weights and static pressure actuators, the actual mean value of the exciting force is not 0 kN, so the mean value of the sample force is not 0 kN.

The exciting force passes through the resonant system to the sample force, and then Equations (15) and (16) are obtained.

(15) $$F_{s}=hF_{e}$$

(16) $$h=\frac{F_{s}}{F_{e}}=\frac{948.25}{10}\approx 94.8$$

#### 3.3.2. Displacement and Phase Analysis

**Figure 9:**
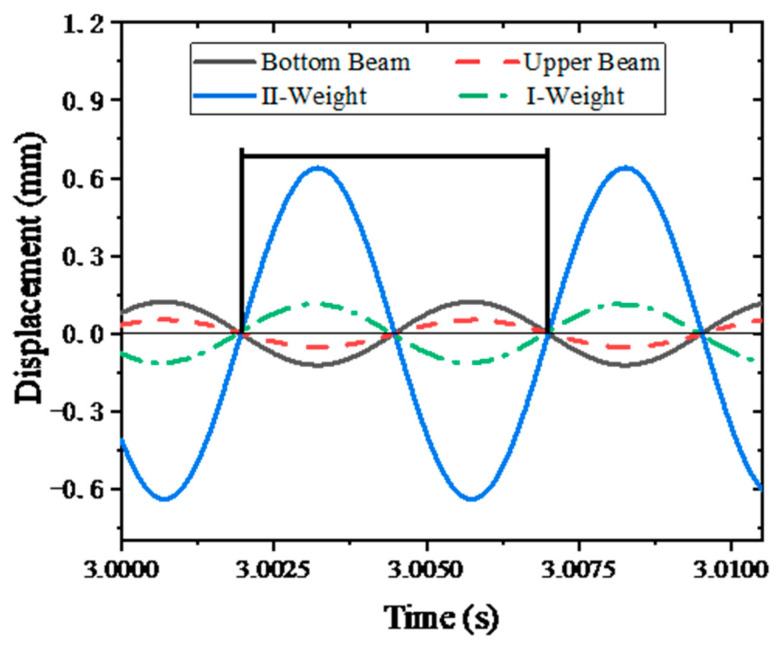
Motion displacement curve.

According to the simulation results, the motion displacement of the lower beam, upper beam, class I weight, and class II weight were analyzed, as shown in Figure 9. The motion amplitude of class II weight is the largest, at 1.27894 mm. The motion amplitude of the upper beam is the smallest, at 0.10572 mm. The motion amplitude of the lower beam is 0.2445 mm, and the motion displacement of the class I weight is 0.22898 mm. The lower beam, upper beam, I weight, and class II weight moving nodes are the same, where class I weight and class II weight phase are the same, and the lower beam and the upper beam phase are the same, and the difference between the two is 180°.

#### 3.3.3. Bearing Capacity Analysis

**Figure 10:**
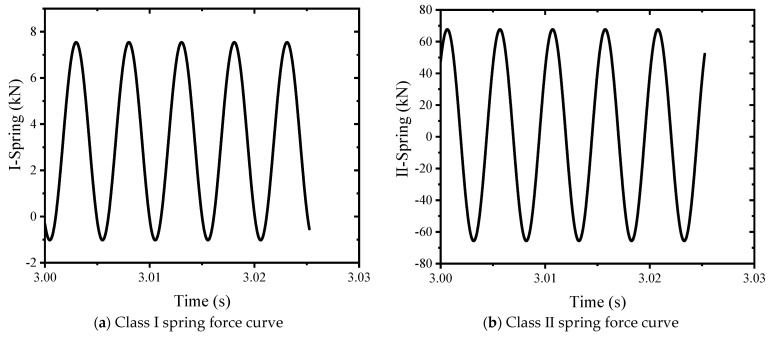

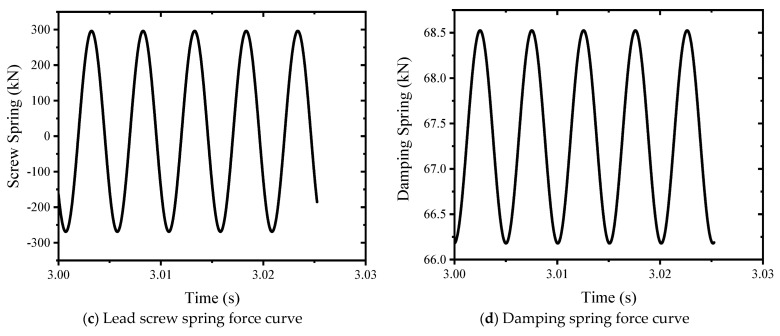
Spring force curve.

According to the simulation results, the motion-bearing capacity of the class I spring, class II spring, lead screw, and shock absorber spring is analyzed, as shown in Figure 10. The half amplitude of the class I spring force is 4.28 kN, the half amplitude of the class II spring force is 66.67 kN, the lead screw force is 282.66 kN, and the force amplitude of the shock absorber spring is 2.3443 kN. Compared with the sample force, the force of the lead screw is much less than the test force, and the resonant loading has low requirements on the frame. This meets the design requirements.

### 3.4. Influencing Factor Analysis of Natural Frequency and Amplification Factor

The natural frequency and amplification factor of the system are the two main parameters for the design of an electro-hydraulic servo resonance system. This section analyzes the influence of sample stiffness, lead screw stiffness, II spring stiffness, II weight mass, lower beam mass, and upper beam mass on the natural frequency and amplification factor of the system.

#### 3.4.1. Influence of Specimen Stiffness

**Figure 11:**
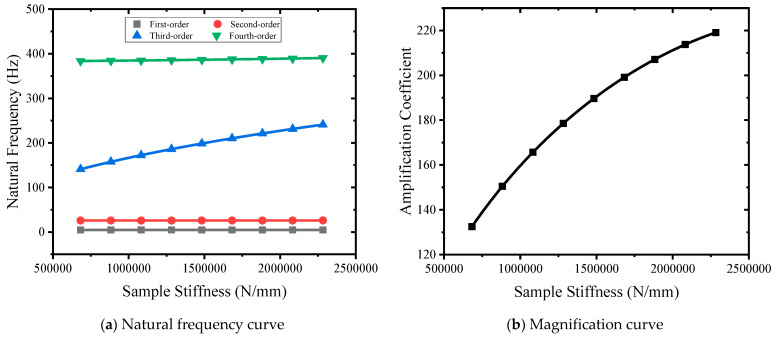
Stiffness curves of different samples.

Using the rod sample with a diameter of 30 mm and standard distance segment length of 150 mm as reference, nine stiffness samples of 682,619 N/mm, 882,619 N/mm, 1,082,619 N/mm, 1,282,619 N/mm, 1,482,619 N/mm, 1,682,619 N/mm, 1,882,619 N/mm, 2,082,619 N/mm, and 2,282,619 N/mm were selected for simulation analysis. The natural frequency–specimen stiffness curve is shown in Figure 11a. The specimen stiffness changes from small to large, and the first-order natural frequency remains unchanged. The second-order natural frequency becomes larger, from 25.76 Hz to 25.9 Hz. The third-order natural frequency becomes larger, from 141.01 Hz to 241.08 Hz. The fourth-order natural frequency becomes larger, from 383.75 Hz to 390.46 Hz. The stiffness of the sample mainly affects the third-order natural frequency. As shown in Figure 11b, the sample stiffness curve changes from small to large, and the magnification coefficient changes from 132.4 to 219, showing a nonlinear increase.

#### 3.4.2. Influence of Lead Screw Stiffness

**Figure 12:**
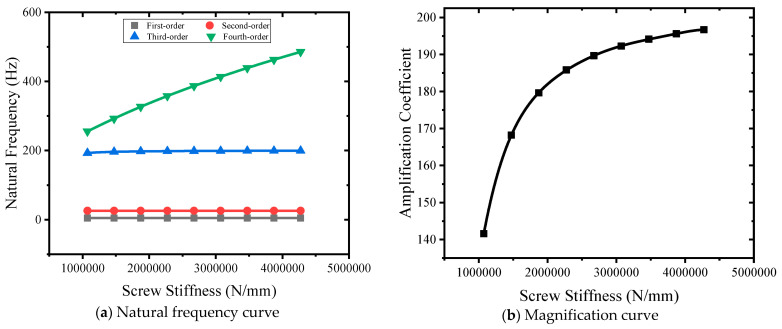
Stiffness curves of different lead screws.

Nine kinds of lead screw stiffness of 1,072,750 N/mm, 1,472,750 N/mm, 1,872,750 N/mm, 2,272,750 N/mm, 2,672,750 N/mm, 3,072,750 N/mm, 3,472,750 N/mm, 3,872,750 N/mm, and 4,272,750 N/mm were selected to carry out simulation points. The natural frequency–specimen stiffness curve is shown in Figure 12a. The lead screw stiffness changes from small to large, and the first-order natural frequency remains unchanged. The second-order natural frequency is unchanged. The third-order natural frequency becomes larger, from 192.89 Hz to 199.36 Hz. The fourth-order natural frequency becomes larger, from 255.28 Hz to 485.39 Hz. The stiffness of the specimen mainly affects the fourth-order natural frequency. As shown in Figure 12b, the sample stiffness curve changes from small to large, and the magnification coefficient changes from 141.56 to 196.69, showing a nonlinear increase.

#### 3.4.3. Influence of II Spring Stiffness

**Figure 13:**
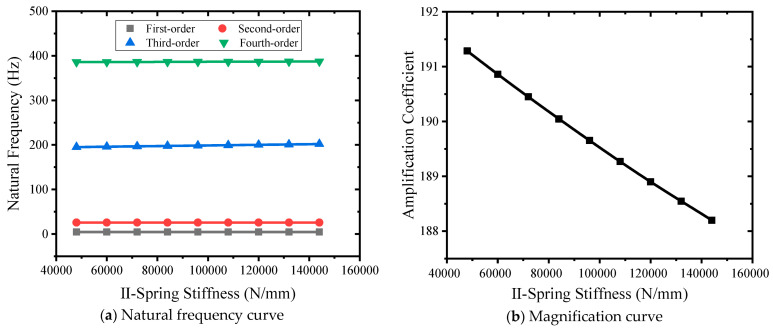
Different II spring stiffness curves.

Nine kinds of class II spring stiffness, including 48,000 N/mm, 60,000 N/mm, 72,000 N/mm, 84,000 N/mm, 96,000 N/mm, 108,000 N/mm, 120,000 N/mm, 132,000 N/mm, and 144,000 N/mm, were selected to carry out simulation analysis. The natural frequency-grade II spring stiffness curve is shown in Figure 13a. The stiffness of grade II spring changes from small to large, and the first-order natural frequency remains unchanged. The second-order natural frequency is unchanged. The third-order natural frequency becomes larger, from 195.17 Hz to 201.97 Hz. The fourth-order natural frequency becomes larger, from 385.82 Hz to 387.26 Hz. The secondary spring stiffness has little effect on the natural frequency. As shown in Figure 13b, the stiffness curve of the class II spring changes from small to large, and the magnification coefficient changes from 191.28 to 188.19, which is close to a linear decrease.

#### 3.4.4. Influence of the Quality of Class II Weights

**Figure 14:**
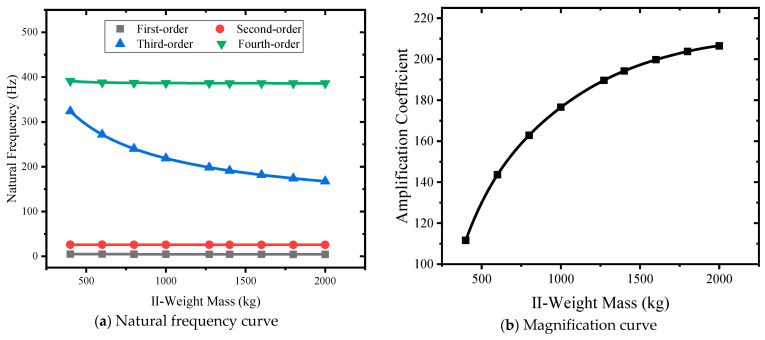
Different II weights mass curves.

Nine kinds of II weight masses of 400 kg, 600 kg, 800 kg, 1000 kg, 1272 kg, 1400 kg, 1600 kg, 1800 kg, and 2000 kg were selected to carry out simulation analysis. The quality curve of natural frequency-II weight is shown in Figure 14a, and II weight mass changes from small to large, and the first-order natural frequency decreases from 5.01 Hz to 4.46 Hz. The second-order natural frequency becomes larger, from 26.11 Hz to 25.71 Hz. The third-order natural frequency becomes smaller, from 323.95 Hz to 167.67 Hz. The fourth-order natural frequency becomes smaller, from 391.49 Hz to 386.23 Hz. The mass of the second-order weight mainly affects the third-order natural frequency. As shown in Figure 14b, the mass curve of class II weights changes from small to large, and the amplification factor changes from 141.56 to 196.69, showing a nonlinear increase.

#### 3.4.5. Influence of Lower Beam Quality

**Figure 15:**
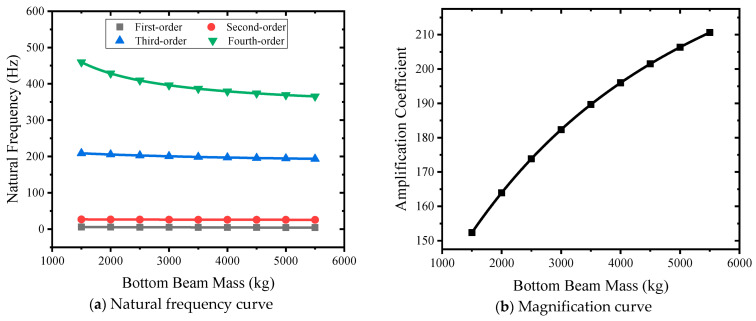
Different lower beam mass curves.

Nine types of lower beam masses of 1500 kg, 2000 kg, 2500 kg, 3000 kg, 3500 kg, 4000 kg, 4500 kg, 5000 kg, and 5500 kg were selected to carry out simulation analysis. The natural frequent and lower beam mass relation curve is shown in Figure 15a. The mass of the lower beam changes from small to large, and the first-order natural frequency decreases from 5.5 Hz to 4.13 Hz. The second-order natural frequency becomes smaller, from 36.7 Hz to 25.44 Hz. The third-order natural frequency becomes smaller, from 208.64 Hz to 193.44 Hz. The fourth-order natural frequency becomes smaller, from 459.59 Hz to 3653.35 Hz. The mass of the lower beam mainly affects the third-order natural frequency. The mass curve of the lower beam is shown in Figure 15b. The mass of the lower beam changes from small to large, and the amplification factor changes from 141.56 to 196.69, showing a nonlinear increase.

#### 3.4.6. Influence of the Upper Beam Quality

**Figure 16:**
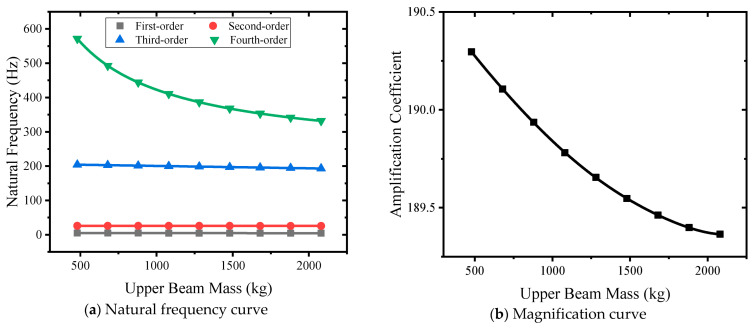
Different upper beam mass curves.

Nine kinds of upper beam masses of 480 kg, 680 kg, 880 kg, 1080 kg, 1280 kg, 1480 kg, 1680 kg, 1880 kg, and 2080 kg were selected to carry out simulation analysis. The natural frequent-upper beam mass curve is shown in Figure 16a. The mass of the upper beam increases from small to large, and the first-order natural frequency decreases from 4.98 Hz to 4.44 Hz. The second-order natural frequency becomes smaller, from 26.12 Hz to 25.66 Hz. The third-order natural frequency becomes smaller, from 204.3 Hz to 193.15 Hz; The fourth-order natural frequency becomes smaller, from 571.47 Hz to 331.89 Hz. The mass of the upper beam mainly affects the third-order natural frequency. The mass curve of the upper beam is shown in Figure 16b. The mass of the lower beam changes from small to large, the amplification factor changes from 190.29 to 189.36, and the nonlinearity decreases. This meets the design requirements.

In summary, aiming at the proposed electro-hydraulic servo resonance technology, the simulation model of the electro-hydraulic servo resonance system is established. Through modal analysis and vibration analysis, the natural frequencies and corresponding modal vectors of the four systems are obtained, the system magnification is calculated, and the feasibility of the proposed technology is verified. Electro-hydraulic servo resonance technology realizes the loading of large tonnage and high-frequency sound by small load and high-frequency sound excitation, which meets the requirements of test loading.

## 4. Conclusions

The paper designs the electro-hydraulic servo resonance system for dynamic and static loading, resonant loading, and electro-hydraulic servo excitation structure. The vibration equation of the system with four degrees of freedom is established, and its vibration characteristics are analyzed. The system model is created using the simulation method, and modal analysis and motion analysis are conducted. The fourth-order natural frequency and corresponding mode vector of the system are solved. Based on the mode vector, the effective excitation frequency of the electro-hydraulic servo is determined as the third-order natural frequency. The impact of sample stiffness, lead screw stiffness, class II spring stiffness, class II weight mass, upper beam mass, and lower beam mass on the natural frequency and amplification factor of the system is analyzed. The results indicate that the electro-hydraulic resonance test technology is feasible. The sample stiffness and class II weight mass primarily affect the third-order natural frequency, while the lead screw stiffness primarily affects the fourth-order natural frequency. The stiffness of the sample, lead screw, class II weight mass, lower beam mass, and upper beam mass have a positive effect on the natural frequency and amplification factor, whereas the stiffness of the class II spring and the amplification factor have a negative effect. The research on electro-hydraulic servo resonance technology expands the loading capacity of fatigue tests and provides a theoretical foundation for the development of high-frequency noise fatigue test equipment for large tonnage.

## Figures and Tables

**Table 1:** Model parameter settings.

Serial	Name	Value	Number	Serial	Name	Value	Number
1	$m_{1}$ (kg)	816	1	6	k_1_′ (N/mm)	3880	2
2	$m_{2}$ (kg)	1272	1	7	k_2_ (N/mm)	16,000	6
3	$m_{3}$ (kg)	1280	1	8	k_3_ (N/mm)	2,672,750	2
4	$m_{4}$ (kg)	5500	1	9	k_4_ (N/mm)	1,482,819	1
5	*k_h_* (N/mm)	11,292	1	10	k_5_ (N/mm)	1000	6

**Table 2:** Natural frequency.

Natural Frequency	Value Hz
ω_1_	4.69
ω_2_	25.86
ω_3_	198.62
ω_4_	386.54

## Data Availability

The authors confirm that the data supporting the findings of this study are available within the article.
